# What Explains Child Malnutrition of Indigenous People of Northeast India?

**DOI:** 10.1371/journal.pone.0130567

**Published:** 2015-06-29

**Authors:** Konsam Dinachandra Singh, Manoj Alagarajan, Laishram Ladusingh

**Affiliations:** 1 Department of Development Studies, International Institute for Population Sciences, Mumbai, India; 2 Department of Development Studies, International Institute for Population Sciences, Mumbai, India; 3 Department of Mathematical Demography and Statistics, International Institute for Population Sciences, Mumbai, India; Örebro University, SWEDEN

## Abstract

Household risk factors affecting child health, particularly malnutrition, are mainly basic amenities like drinking water, toilet facility, housing and fuel used for cooking. This paper considered the collective impact of basic amenities measured by an index specially constructed as the contextual factor of child malnutrition. The contextual factor operates at both the macro and micro levels namely the state level and the household level. The importance of local contextual factors is especially important when studying the nutritional status of children of indigenous people living in remote and inaccessible regions. This study has shown the contextual factors as potential factors of malnutrition among children in northeast India, which is home to the largest number of tribes in the country. In terms of macro level contextual factor it has been found that 8.9 per cent, 3.7 per cent and 3.6 per cent of children in high, medium and low risk households respectively, are severely wasted. Lower micro level household health risks, literate household heads, and scheduled tribe households have a negating effect on child malnutrition. Children who received colostrum feeding at the time of birth and those who were vaccinated against measles are also less subject to wasting compared to other children, and these differences are statistically significant.

## Introduction

Globally 52 million children under 5 years are wasted and 70 percent of them are in Asia [1]. Worldwide, 5 million children die every year due to malnutrition, of which 2 million are from India [2]. This is despite the fact that improvement in water, sanitation and hygiene (WASH) can reduce stunting among children [3], and has been shown to have resulted in height increase among children much more than other nutritional interventions [4]. The benefit of WASH interventions in protecting against malnutrition is also reported from a number of studies [5–10]. A comparison of preschool children from India and Guatemala indicates that poor levels of sanitation are the main cause of the high level of child malnutrition [11]. Solid fuel use has been suggested as a risk factor for stunting [12] as it causes indoor air pollution—a factor highly conducive to childhood diseases. Household allocation of scarce family resources too affects child health [13]. Children born with low birth weight are more likely to be exposed to the risk of experiencing malnutrition as they grow up [14–15], and are also likely to have high mortality risk [16]. Malnutrition may even cause mental retardation and cerebral palsy [17]. The association between live attenuated measles vaccine and malnourishment is found to be positive [18], though adverse nutritional effects of measles are experienced by all children. Regardless of residence background, the likelihood of stunting increases with age, but no such evidence is found with regard to underweight and wasting [19–20].

About 53 per cent of India’s population defecates in the open [21], exposing children to faecally-transmitted infections. The ‘Asian enigma’ [22], the phenomenon of shorter height of Indian children, is attributed to the lack of access to toilets, and the stunting of children to open defecation [23]. Maternal weight or body mass index (BMI), and excessive vomiting during pregnancy are significant risk factors of child malnutrition [24]. Poor nutritional status of the mother, leading to the child having a low birth weight, further increases the child’ssusceptibility to infections, and aggravates the situation [25]. Christian and Sikh children have relatively better nutritional status than Hindu and Muslim children, but there is no statistically significant difference [26]. Undernutrition is a significant health problem among tribal children, and is associated with the literacy status of mothers, household wealth index, and morbidities [26–27]. There are mixed findings with regard to the association between the sex of a child and malnutrition—no association [28], higher malnutrition among girls as they were fed less than boys [29] and girls having an edge over boys in the case of NE India [30]. Literature available on child malnutrition in India has considered a few of the aforesaid risk factors [30–34]. However these studies have not considered the household environmental health risks as a potential determinant of child malnutrition.

This study emphasizes that the household environment in which a child is born and brought up is an important determiningfactor of child health, more so in the context of the isolated and inaccessible areas of northeast (NE) India. The region comprises eight small states, namely, Assam, Arunachal Pradesh, Manipur, Meghalaya, Mizoram, Nagaland, Sikkim and Tripura. Northeast India is not only physically isolated from the rest of the country due to mountainous terrain and poor infrastructure,it also has diverse socio-cultural practices of bringing up children,given that the region isinhabited by numerous tribal and ethnic groups. These indigenous people live in traditional,thatched roof huts, without basic amenities. The lack of basic amenities isdirectly or indirectly responsible for the prevalence of high child morbidities and malnutrition in this region.

Table 1 shows the socio-demographic profiles of the eight north-eastern states. All these states, except Tripura, have a growth rate higher than the national average. They also have asymmetric demographic profiles. Meghalaya reports the highest and Tripura the lowest natural growth rate of 16.6 and 9.9 per 1000 population respectively. Arunachal Pradesh has the highest and Sikkim has the lowest 0–5 year population constituting respectively 29 per cent and 5 per cent of the total population. The female literacy rates are higher than the national average in all the states except for Arunachal Pradesh. According to Sample Registration System 2011, infant mortality rates (IMR) were found to be lower than the country’s average of 47 deaths per 1000 live births in all the states except Assam (58) and Meghalaya (55).

**Table 1:** Profiles and demographic characteristics of states in northeast India.

	Arunachal Pradesh	Assam	Manipur	Meghalaya	Mizoram	Nagaland	Sikkim	Tripura
Land Area (sq. km.)^1^	83743	78438	22327	22429	21081	16579	7096	10486
Total population in million^1^	1.38	31.21	2.57	2.97	1.10	1.98	0.61	3.67
Population size—% of national population^1^	0.1	2.6	0.2	0.2	0.1	0.2	0.1	0.3
0–5 (population in million) ^1^	0.18	3.94	0.32	0.49	0.15	0.24	0.05	0.39
0–5 (% of state population) ^1^	29.03	12.62	15.96	16.43	5.08	17.55	4.82	10.66
Female literacy rate (%)^1^	57.7	66.3	72.4	72.9	89.3	76.1	75.6	82.7
Population density ^1^	17	398	115	132	52	119	86	350
Sex ratio—Females per 1000 males^1^	938	958	992	989	976	931	890	960
0–5 Sex ratio—Females per 1000 males^1^	970	963	930	972	971	943	953	961
Birth rate^2^	20.5	23.2	14.9	24.5	17.1	16.8	17.8	14.9
Death rate^2^	5.9	8.2	4.2	7.9	4.5	3.6	5.6	5
Natural growth rate^2^	14.6	14.9	10.7	16.6	12.5	13.2	12.3	9.9
Infant mortality rate^2^	31	58	14	55	37	23	30	27

*Sources*: 1- Census of India, 2011, 2- Sample Registration System, 2011

As stated earlier,the purpose of this study is to understand the effect on child malnutrition of the composite household risk due to lack of safe drinking water, lack of toilet and drainage facilities, poor housing conditions, and indoor air pollution due to use of biomass fuel for cooking. Also,underprivileged ethnic groups in remote areas are more at risk of severe malnourishment [35–36]due to limited access to healthcare, poor dietary habits, poor hygiene, and above all, poverty. It is for these reasons that the NE is an important case for the present study.

It is an accepted fact that child malnutrition is the outcome of multiple risk factors. This suggests the need to integrate a host of correlates at the levels of the child, mother, household and state with the composite household risk to explain child malnutrition. The sex of a child is important in the context of discrimination againstthe girl child in access to intra-household resources particularly food, healthcare and education. Studies foundthat age and sex of child, age of the mother, occupation, education, parity, and number of siblings havean effect on the prevalence of child malnutrition (stunting, wasting and underweight) [20, 37–39]

The foregoing paragraphs have highlighted some of the household contextual factors, and somechild, woman and household characteristics thathave either a positive or a negative association with child malnutrition. Three points may be noted at this juncture. First, most of the findings do not pertain to NE India, and it is not certain whether theyare applicable tothis region or not—this needs to be validated. Second, there is no evidence of an integration ofthe composite household risk, and the child, mother and household characteristics in the assessment of factors affecting malnutrition. Third, the household context has been either overlooked, or considered on a piecemeal basis in most of the aforesaid studies that have attempted to identify detrimental factors associated with child nutrition. This paper is an attempt to enrich the understanding of the extent of child malnutrition in NE India and its associated factors in the light of these points. The merit of the study can be assessed from the fact that from 1998–99 to 2005–06,severe wasting in NE India increased from 2 to 7 per cent, severe stunting from 17 to 19 per cent, and severe underweight from 7 to 11 per cent [40].

## Data and Methods

### Ethics Statement

The study is based on data available in the public domain, therefore no ethical issue is involved.

### Data

The Census of India is a rich source of information on basic household amenities, such as, drinking water facility, toilet and bathroom facility, cooking fuel, electricity, drainage system, separate rooms for kitchen and living, and housing conditions. Basic household amenities are available for all households by states, districts and villages/urban units. Data on these basic amenities from Census 2001 and 2011 were used to constructa macro amenity index (MAI)for the states in NE India. Census is conducted in India every 10 years. The first census was conducted in 1872 and the latest in 2011.

Unit level data on the nutritional status of children, child and maternal characteristics and household provision for drinking water, toilet facility, type of cooking fuel and housing condition are taken from the third round of the National Family Health Survey, 2005–06 (NFHS-3). The NFHS-3 is a nationwide survey of maternal and child health including nutritional status and also provides data on domestic violence and HIV incidence. It covered 124,385 women in the age group 15–49 years and 74,369 men in the age group15–54years and adopted a multi-stage stratified design.

### Measures

Two indices, macro amenity index (MAI) and household environment health risk index (HEHRI), were constructed to measure lack of amenities in the households at state level and health risk factors at the household level respectively.

Macro amenity index (MAI): Lack of amenities at the community and state level lead to diseases which are the leading cause of major morbidities and mortality among children in developing countries, especially in rural areas. Many researchers [41–44]have found that indoor environment play a significant role in child health and child survival. In this study, in order to objectively quantify household environmental health risks at the state level, MAI is constructed using data from Census 2001 and 2011.

Amenities included in the construction of MAI are number of rooms, house type, toilet and bathroom facilities, drainage system, lighting provision, provision for kitchen, type of fuel used and distance to source of drinking water from household. Percentages of households without these amenities are computed at the state level and on the basis of these percentages for each of the amenities,the states are assigned scores of 0, 1 or 2. Percentages ranging from 0–20 are assignedscore 0, 21–50 are assigned score 1 and 51–100 are assigned score 2. MAI is constructed by adding these scoresat the state level. The numerical value of MAI ranges from 5 to 10. Household amenities at state level is categorized as high if the MAI score is 5–6,categorized as medium amenities if MAI score 7–8 andcategorized as low amenities if the MAI score is 9–10. The purpose of categorization of MAI is to have a comprehensive knowledge of the association between child malnutrition and contextual background of health related amenities at state level.

Household Environment Health Risk Index (HEHRI): Environmental risk index represents a reasonable aggregate measure that can be used to compare environmental risks for households in a given community or from one community to another [45]. In order to objectively quantify the household environment health risks, HEHRI is constructed. Four main critical risk or protective factors are used. HEHRI focuses on the risk factors present in the living space utilized by the entire household. Those risk or protective factors are source of drinking water, type of toilet facility, cooking fuel used and type of house. Each of the factors is assigned a relative score based on the association with environmental health problems of children.,Household provision for drinking water, toilet facility, cooking fuel and type of house are assessed from NFHS-3 and coded as 1 wherever there is provision and otherwise, 0. HEHRI is the sum of the scores for these lifesaving amenities at the household level. Households with HEHRI score 3–4 are categorized as low health risk; 1–2 as medium health risk and households with 0 score are categorized as high health risk.

### Outcome variable

Child malnutrition measured by weight-for-height is considered as dependent variable for this study. Low weight-for-height (wasting or thinness) indicates recent and severe weight loss due to acute starvation and/or severe disease. It may also be the result of chronic unfavourable conditions. Wasting can be defined as the percentage of children whose weight-for-height is less than -2SD below the median [46] and similarly severe wasting is the proportion of children whose weight-for-height is less than -3SD below the median (globally accepted cut-off, WHO 1995). The measure is expressed in the form of z-scores standard deviation (SD) from the median of the 2006 WHO International Reference Population [47].

Wasting is usually uncorrelated with height-for-age (stunting) and 20 per cent variability of weight-for-age (underweight) is accounted. Wasting in children is a symptom of acute malnutrition (under-nutrition), usually as a consequence of poor dietary intake or high incidence of infectious diseases, especially diarrhoea and vice versa. Undernourished children have poor immune system which increases the severity and duration of, and susceptibility to, infectious diseases, and an increased risk of death.

A healthy child gains 2 to 3 kilograms each year, failing which it runs the risk of wasting. This may be due to poor dietary intake [48]. In the northeastern states of India, more than 8 per cent of children under age 5 are moderately wasted and more than 6 per cent are severely wasted (less than -3SD). This is more than the acceptable range of below 5 per cent [49–50] as defined by the World Health Organization. The source of outcome variable for this study is the NFHS-3.

### Explanatory variables

A number of socio-demographic factors contribute to differentialsin child malnutrition. Based on the review of determinants of child malnutrition highlighted in the introduction section and considering the relevance in the context of NE India in this study,potential factors included for the child areage, sex, colostrum feeding and measles vaccination. Age of mother, exposure to mass media, child death experience, working status and BMIare included for the mother’s background,while at household level,the factors are place of residence, caste or tribe and literacy status of head of the household. All explanatory variables used in the analysis are from NFHS-3.

### Analysis

Graphical representation is used to show the distribution of children NE India by levels of MAI and prevalence of child wasting by levels of HEHRI. Cross tabulation and descriptive statistics are used to show prevalence of child wasting by selected household, child and maternal characteristics.

To disentangle the hierarchical data collected from multi-stage sampling design, and interdependence of children on women and women on household a three-level normal regression was used for multivariate analysis. Random intercept multilevel regression is defined as follows:

$$\begin{array}{l} y_{ijk}=\beta_{0ijk}+\beta_{1}x_{ijk}\\ \beta_{0jk}=\beta_{0}+v_{0k}+u_{0jk}+e_{0ijk} \end{array}$$

wherey_ijk_ is the z-score of weight-for-height of i^th^ child of j^th^ woman in the k^th^ household and x_ijk_ are the corresponding explanatory variables. β_0jk_ is the random intercept, β_1_ s are regression coefficients of x_ijk_.

*v*_0k_ is the random effect at the household level, an allowed-to-vary departure from the grand mean;

*u*_0jk_ is the random effect at the mother level, a departure from the household effect;

*e*_0ijk_ is the random effect at the child level, a departure from the mother effect within a household

MS-Excel, STATA 12 and MLwiN 2.02 were used for analysis.

## Results

### Basic amenities and child wasting

**Fig 1:**
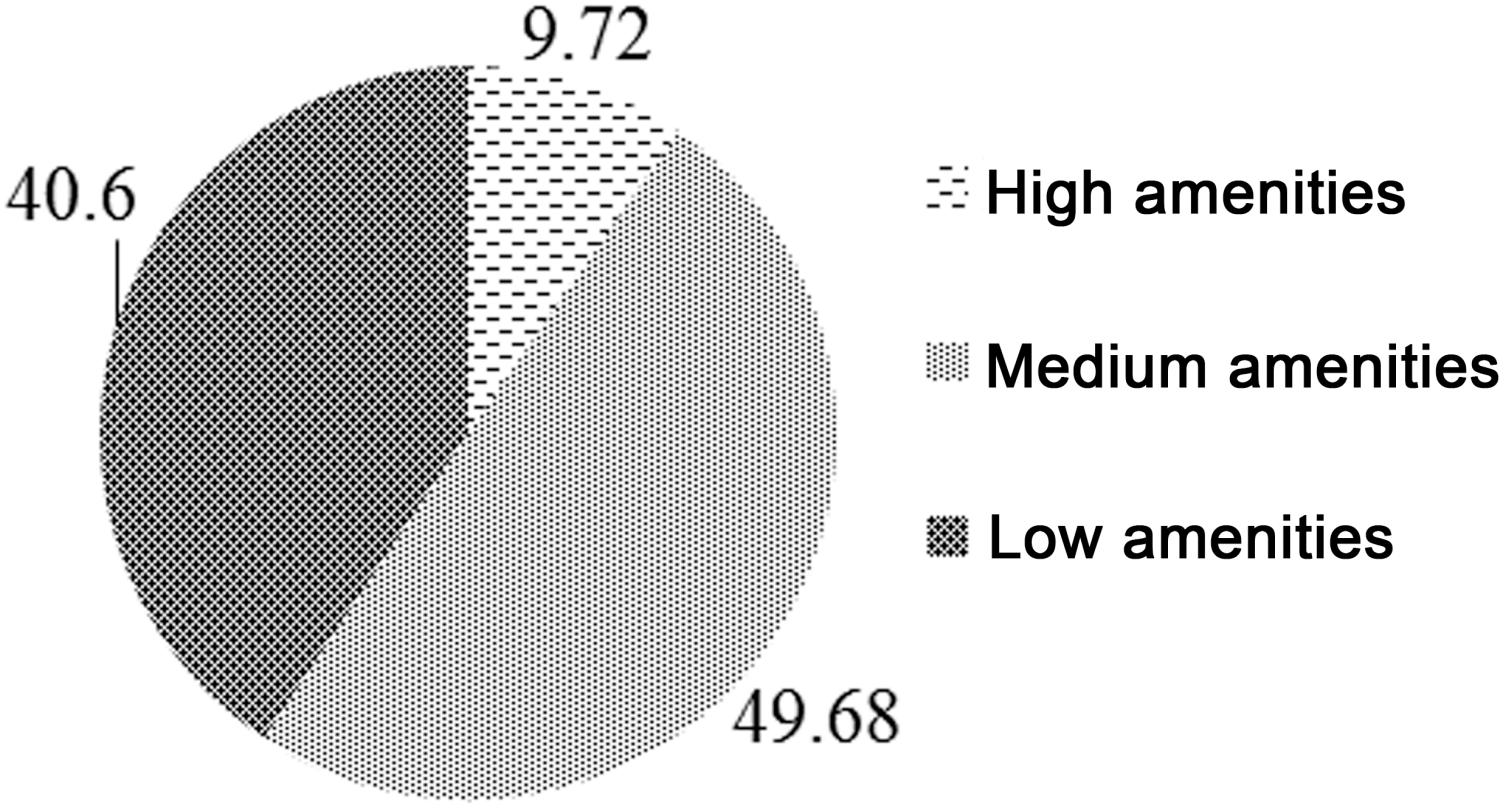
Children in 0–59 months by level of Macro Amenities Index (MAI) in Northeast India. Source: Based on authors computation from Census 2001–2011 and NFHS-3 (2005–06)

The lack of basic amenities in states of NE India can be assessed by looking at the distribution of children in the age group 0–59 months by level of amenities shown in Fig 1. It can be seen 40 per cent children are from households with low amenities, 50 per cent from households with medium amenities and only 10 per cent are from households with high amenities on the scale of MAI.

**Fig 2:**
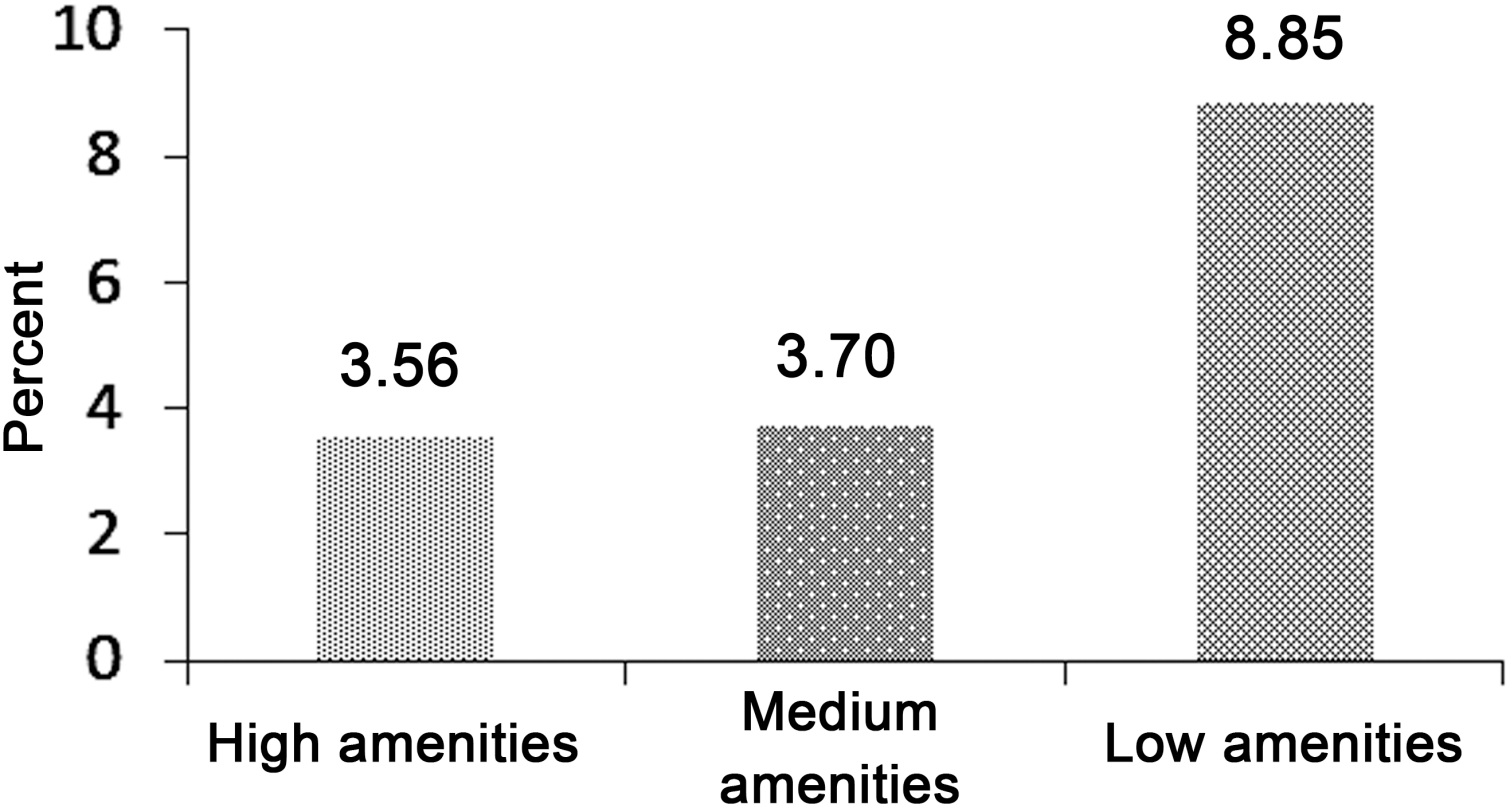
Prevalence of severe wasting of children 0–59 months by Macro Amenities Index (MAI) in Northeast India. Source: Based on authors computation from Census 2001–2011 and NFHS-3 (2005–06)

Fig 2 provides a graphical representation of the association between the prevalence of wasting among children under5 years and the level of MAI in NE India. It is found that the prevalence of severe wasting is highest among children from low MAI households and lowest for those children from high MAI households. Severe wasting among children from low, medium and high MAI households are 3.6, 3.7 and 8.9 per cent respectively.

### Prevalence of child wasting

Table 2 provides cross tabulation of the extent of severe wasting of children and household background, parental and child characteristics. The prevalence of severe wasting is as high as 6 per cent in rural areas,7 per cent among scheduled tribes (STs), and 8 per cent in householdswith illiterate heads. In households with a highHEHRI,7 per cent of children are severely wasted, while in households with a low HEHRI,only 5 per cent of children are severely wasted.

**Table 2:** Prevalence of severe wasting among children 0–59 months in northeast India by selected household background, parental and child characteristics, 2005–06.

Variable	Nominal Categories	Sample (%)	Severe Wasting (%)	Std. Deviation
***Household Characteristics***				
Place of residence	Urban	32.5	4.29	0.54
	Rural	67.5	6.17	0.47
Caste/tribe	Scheduled Caste	39.1	4.45	0.47
	Scheduled Tribe	51.6	6.81	0.62
	Others	9.3	5.04	1.09
Head literacy	Illiterate	25.6	8.12	0.81
	Literate	74.4	4.86	0.35
Household health risk	High	34.3	7.31	0.73
	Medium	46.7	4.84	0.47
	Low	18.9	4.67	0.64
***Mother's Characteristics***				
Age	15–19	4.4	7.42	1.49
	20–29	56.6	6.13	0.48
	30–39	34.4	4.84	0.47
	40–49	4.6	6.86	1.68
Mass media exposure level	None	17.6	8.89	0.96
	Low	37.5	6.17	0.60
	Medium	32.7	4.56	0.49
	High	12.2	2.21	0.48
Child death experience	No	84.3	5.65	0.40
	Yes	15.7	6.35	0.87
Working status of woman	Not working	58.4	5.82	0.45
	Non Agricultural	18.3	4.21	0.66
	Agricultural	23.3	6.68	0.86
Body Mass Index	Underweight	21.0	6.88	0.72
	Normal	70.9	5.67	0.43
	Overweight	8.1	3.39	0.75
***Child Characteristics***				
Sex of Child	Boy	49.8	6.02	0.51
	Girl	50.2	5.52	0.42
Age group in months	0–6	9.9	9.00	1.04
	7–12	9.9	7.46	0.97
	13–24	19.7	5.99	0.77
	25–36	21.2	5.05	0.57
	37–59	39.3	4.77	0.54
Measles	No	58.1	6.60	0.49
immunization	Yes	41.9	4.51	0.47
Colostrum given	No	19.8	6.50	0.71
	Yes	80.2	5.60	0.41
**Total number of children**		**7574**		

*Source*: Based on authors’ computation from NFHS-3 (2005–06)

Children of mothers aged 15 to 19 years have the highest percentage of severe wasting, while children of mothers who do not experience child death and are exposed to mass media are less likely to suffer from severe wasting. The prevalence of severe wasting among children of mothers who work in agriculture, and among those whose mothers are underweight is 7 per cent each. The prevalence of severe wasting is higher among boys than girls, and it is as high as 9 per cent for children under 12 months. For children who were not breastfed with colostrum, and those who were not immunized against measles, the prevalence of severe wasting is 7 per cent.

**Fig 3:**
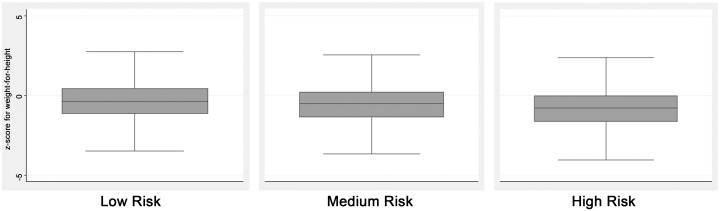
Box plot of z-score of weight-for-height of children in 0–59 months by level of Household Environment Health Risk Index in Northeast India. Source: Based on authors computation from National Family Health Survey 3 (NFHS-3), 2005–06

The box plot of z-score for weight-for-height by levels of HEHRI depicts the impact of household health risk on child malnutrition (Fig 3). It can be seen that the median of z-scores for weight-for-height decreases with increasing level of HEHRI suggesting the disadvantage faced by children in households with inadequate basic amenities as far as wasting is concerned.

### Determinants of child wasting

**Fig 4:**
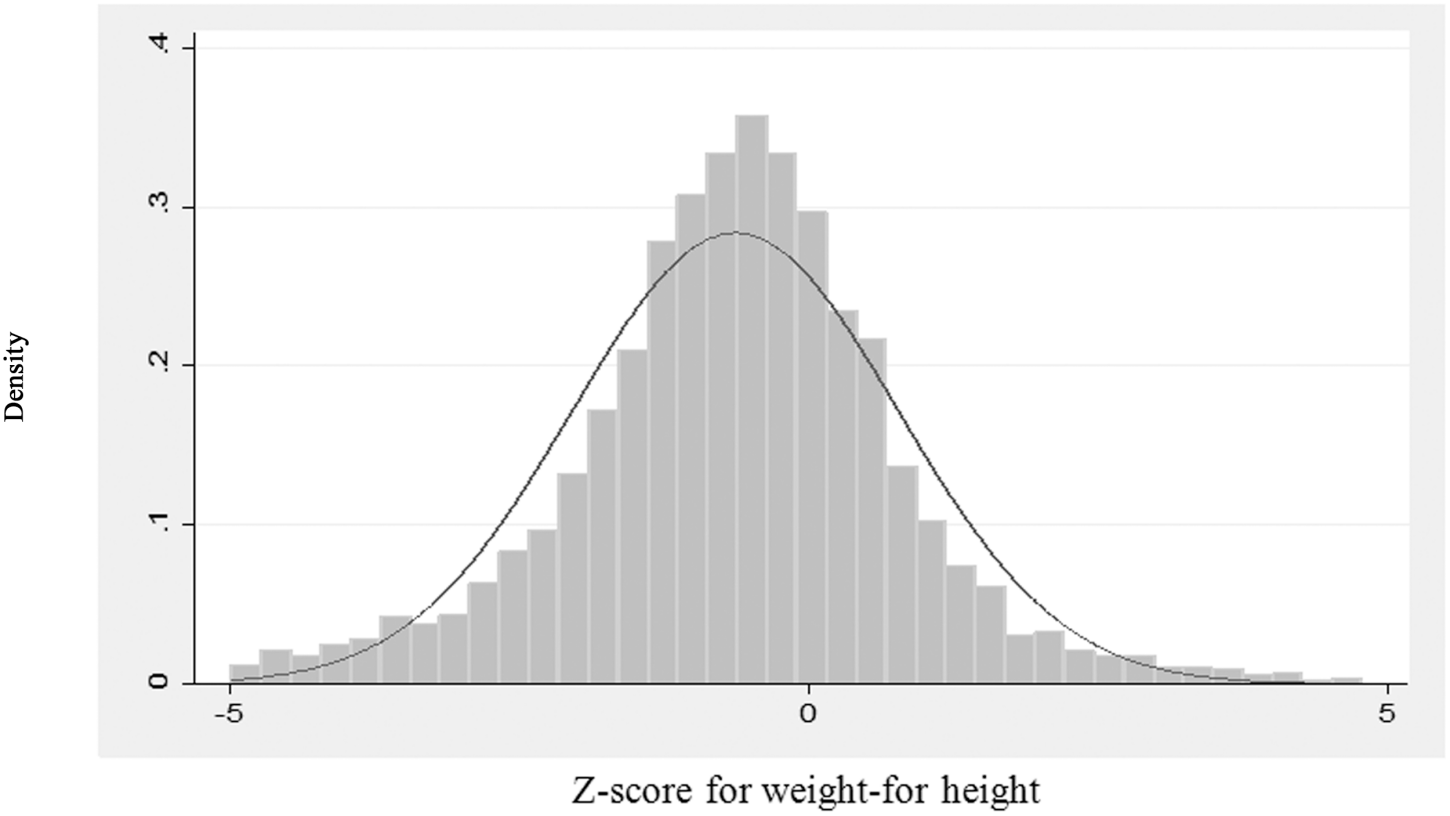
Histogram and normal density plot of z-score of weight-for-height of children in 0–59 months in Northeast India. Source: Based on authors computation from National Family Health Survey 3 (NFHS-3), 2005–06

To find significant determinants of the nutritional status of children, the z-score for weight-for-height is taken as the dependent variable in multilevel regression. Use of multilevel regression is justified considering hierarchical structure of children living in households, and households in villages or urban localities. To choose an appropriate method for multivariate analysis, the histogram of the z-score for weight-for-height is plotted with density curve (Fig 4). The distribution of z-scores is fairly normal suggesting appropriateness of the use of normal regression.

The dependent variables are the composite household risk measured by the HEHRI, literacy and caste of household head, place of residence, mother’s background including age, education, working status and nutritional status, and child’s characteristics, such as age in months, birth weight, colostrum feeding, and sex of children aged 0 to 59 months. In order to examine the changing nature of explanatory potential of these factors, multilevel regression is implemented in steps considering the subsets of dependent variables in every step, and designated as Model 1, 2, 3 and 4. Estimated coefficients which indicate magnitude and direction of each independent variable on wasting of children while the effect of all other variables are controlled and significance levels are shown in Table 3. The value of the deviance statistic is lowest for Model 4 and this is considered as the final model for explaining adjusted effect of the selected background characteristics on wasting of children in the age group 0–59 months in NE India.

**Table 3:** Estimated effects and significance levels of selected background characteristics on z-score of weight-for-height.

	Model 1	Model 2	Model 3	Model 4
Constant	-0.64(0.02)**	-1.07(0.04)**	-1.36(0.09)**	-1.64(0.11)**
**Place of residence**				
Rural^®^				
Urban		0.10(0.04)*		0.05(0.04)
**Caste/tribe**				
Scheduled Caste^®^				
Scheduled Tribe		0.21(0.04)**		0.22(0.04)**
Other		-0.06(0.06)		-0.02(0.06)
**Household head literacy**				
Illiterate^®^				
Literate		0.15(0.04)**		0.09(0.04)*
**Household health risk**				
High ^®^				
Medium		0.23(0.04)**		0.16(0.04)**
Low		0.38(0.06)**		0.22(0.06)**
**Age of mother (years)**				
20–29^®^				
15–19			0.15(0.08)	0.18(0.08)*
30–39			0.07(0.04)	0.06(0.04)
40–49			-0.05(0.05)	-0.07(0.05)
**Child death experience**				
Yes^®^				
No			0.08(0.05)	0.07(0.05)
**Mass media exposure level**				
None^®^				
Low			0.21(0.05)**	0.16(0.05)**
Medium			0.32(0.05)**	0.22(0.06)**
High			0.26(0.07)**	0.18(0.07)*
**Working status of mother**				
Not working^®^				
Non-agricultural worker			0.03(0.05)	0.02(0.05)
Agricultural worker			-0.02(0.04)	-0.05(0.04)
**Body Mass Index**				
Underweight^®^				
Normal			0.40(0.04)**	0.34(0.04)**
Overweight			0.68(0.07)**	0.60(0.07)**
**Sex of the child**				
Girl^®^				
Boy			0.02(0.03)	0.02(0.03)
**Age of the child (months)**				
0–6 ^®^				
7–12			-0.13(0.07)	-0.13(0.07)
13–24			-0.06(0.06)	-0.08(0.06)
25–36			-0.03(0.06)	-0.04(0.06)
37–59			-0.01(0.06)	0.00(0.06)
**Measles immunization**				
No^®^				
Yes			0.11(0.04)**	0.09(0.04)**
**Colostrum given**				
No^®^				
Yes			0.05(0.04)**	0.09(0.04)*
**Ωu** ^**2**^	0.55(0.04)	0.50(0.04)	0.49(0.04)	0.46(0.04)
**Ωe** ^**2**^	1.43(0.04)	1.43(0.04)	1.42(0.04)	1.43(0.04)
**Deviance**	26520	26341	26271	26184
**Intra-class correlation coefficient**	27.8%	25.9%	25.7%	24.3%

Significant level:

* p< 0.05;

** p< 0.01;

^®^ –reference category

Source: Based on authors’ computation from NFHS-3 (2005–06)

Model 1 is an empty model without any control variable, with a random intercept for communities, measuring variation in aggregate risk of child malnutrition between communities. This model is used as a reference model for assessing the improvement of multilevel models when one subset of independent variables is in the next step of regression.

In addition to a random intercept, Model 2 considers the household variables. The estimates reveal that in NE India,urban householdshavea significant edge over rural households in terms of nutritional status of children. Moreover,in SThouseholds with literate heads, having medium and low HEHRI,there is a positive relation with the nutritional status of children,and the difference is significant at p<0.01.

The parametric estimate of Model 3 indicates that exposure to mass media and the BMI of the mother have a significant effect on the nutritional status of children (p<0.01). At the same time,immunizationof the child againstmeasles(the last vaccine in child immunization),and breastfeeding with colostrum have a positive effect on height-for-weight as indicated by the significance ofz-scorefor p<0.01.

In Model 4 important household factors which have a significant rolein lowering wasting children in the age group 0–59 months are medium and high HEHRI scores, literate household heads, and ST households. In addition, children of mothers aged 15–19 years, exposed to mass media, and not underweight have a lower risk of suffering from wasting; this is statistically different from other children. Children who received colostrum feeding at the time of birth and those who were vaccinated for measles are also less subject to wasting compared to other children, and these differences are statistically significant.

There is high intra-class correlation coefficient of children (24 per cent) indicating clustering of child wasting, that is, the probability that any two children randomly selected from a randomly taken place are both wasted is 24.3 per cent in NE India.

## Discussion and Conclusion

This paper makes an attempt to study the totality of the effect of lack of proper toilet facilities, potable water, cooking fuel and housing conditions by constructing an index of household environment health risk (HEHRI) on child malnutrition in NE India. The region is characterized by high level of household risk with 23 per cent of households lacking provision for potable water, 32 per cent having no toilet, 81 per cent using biomass fuel for cooking, 42 per centof houses either temporary or in dilapidated condition and 74 per cent having no drainage facility (estimated for 2005–06 from Census of India, 2001 and 2011). Household environment health risk index which captured the vulnerability of households in NE India foundthat these factors have a detrimental effect on nutritional status of childrenof the region. Detrimental effect of lack potable water and sanitation on child nutrition was also the finding of other studies [3, 51–54]. Open defecation because of lack of toilet facility in the household exposes children in particular to faecal germs leading to malnutrition as reported by a number of research findings. As in this study,the effect of lack of toilet facility and unhygienic storage of drinking water on child malnutrition was reported in a slum based study in India [55], and merging data from demographic health survey from 1986 to 2007 for 171 countries [56] has provided evidence of lower risks of child death, diarrhoea and stunting associated with access to improved sanitation. It can be concluded assertively that whether it is for the health of children in NE India or elsewhere, access to potable water, toilet facility and sanitation is crucial. Programmes to strengthen accessibility to WASH need to go hand in hand with sustainable community based interventions to spread the message of the dividend on health from good practice of WASH. In the context of communities in remote inaccessible areas of NE India, the educational attainment of the household head and his or her affiliation to a particular tribe or clan plays a role in community engagement and decision making for health care utilization. This is reflected in the findings of this study. Children in households with literate head of household are likely to be healthier than children from households whose heads are non-literate. This is because education serves as catalyst to avoid seeking health-related advice from traditional healers and quacks. This finding reaffirms the importance of educational status of the household head when it comes to important matters such as the health of children, as do the findings of other studies [57–58]. Further,most states in NE India are largely inhabited by STs and are also characterized by diverse healthcare practice and beliefs and feeding practices. Scheduled tribes and scheduled castes have healthier feeding and eating practices while other communities to an extent have food habits more similar to majority of the other Indians. The findings from this study show that children of ST communities have lower levels of wasting than children from other communities.

Well informed mothers, as reflected by exposure to mass media, can have a bearing on the nutritional status of their children and this is supported by the findings of this study which show that children of women exposed to mass media are less likely to be wasted than other children. The importance of mass media exposure of women on their children’s nutritional status is supported bythe findings of a number of studies from elsewhere in India [59–61]. For improving children’s nutritional status in NE India, a social network of women and intensive door-to-door IEC (Information, Education and Communication) activities to educate women on good childcare practices and preserving the nutritional value of foods is the key intervention area.

The study did not find uniform pattern of association of age of women and also child’s age with nutritional status of children. Children’s nutritional status is found to correlate with women’s health as measured by BMI [60, 62] as children of underweight mothers are likely to be more wasted than children of healthy women. There is no significant difference in nutritional status of boys and girls which indicates that there is no discrimination by sex in NE India. Children protected from childhood diseases by proper immunization are found to be healthier in subsequent years than their counterparts who have not been immunized. Feeding colostrum is found to protect children from malnutrition and benefit of colostrum feeding is also supported by existing literature [63–65].

This study not onlyhighlights the vulnerability of children in remote and inaccessible regions but also provides evidence of the need for interventionscollectively involving community, households and parents to eradicate child malnutrition from NE India.
